# Efficient expression of a cnidarian peptide-gated ion channel in mammalian cells

**DOI:** 10.1080/19336950.2021.1882762

**Published:** 2021-02-10

**Authors:** Michèle Bachmann, Audrey Ortega-Ramírez, Lilia Leisle, Stefan Gründer

**Affiliations:** Department of Physiology, RWTH Aachen University, Aachen, Germany

**Keywords:** Codon optimization, Hydra, ligand-gated ion channel, neuropeptide

## Abstract

Hydra Na^+^ channels (HyNaCs) are peptide-gated ion channels of the DEG/ENaC gene family that are directly activated by neuropeptides of the *Hydra* nervous system. They have previously been successfully characterized in *Xenopus* oocytes. To establish their expression in mammalian cells, we transiently expressed heteromeric HyNaC2/3/5 in human HEK 293 and monkey COS-7 cells. We found that the expression of HyNaC2/3/5 using native cDNAs was inefficient and that codon optimization strongly increased protein expression and current amplitude in patch-clamp experiments. We used the improved expression of codon-optimized channel subunits to perform Ca^2+^ imaging and to demonstrate their glycosylation pattern. In summary, we established efficient expression of a cnidarian ion channel in mammalian cell lines.

## Introduction

The degenerin/epithelial Na^+^ channel (DEG/ENaC) gene family consists of Na^+^ channels from multicellular organisms with a variety of gating mechanisms and physiological functions, including peptide-gated ion channels such as Hydra Na^+^ channels (HyNaCs) from the freshwater polyp *Hydra magnipapillata* [1]. *Hydra* belongs to the phylum Cnidaria within the class of Hydrozoa. Thus, it is a sister group to all bilaterian DEG/ENaC-expressing organisms and could enable insight into the evolution of functional characteristics within the channel family.

HyNaCs are obligate heterotrimers [2] and are directly activated by Hydra-RFamide I and Hydra-RFamide II (RFamide I and RFamide II) [1–3], two neuropeptides of the *Hydra* nervous system [4,5]. Electrophysiological properties of HyNaCs have been successfully characterized in *Xenopus laevis* oocytes [1–3,6]. While expression in oocytes typically results in large current amplitudes of the foreign ion channel, expression in mammalian expression systems is better suited to study, for example, trafficking and regulation of an ion channel. In this study, we, therefore, expressed HyNaC2/3/5 [3] in two different mammalian cell lines, HEK 293 and COS-7 cells. We show that codon optimization leads to an efficient expression of HyNaC2/3/5 in HEK 293 cells. To show the utility of this system for the study of HyNaCs, we performed Ca^2+^ imaging and characterized the N-linked glycosylation of HyNaC2/3/5.

## Materials and methods

### Molecular biology

To detect HyNaCs in western blots, we fused three hemagglutinin tags (HA-tags; YPYDVPDYA) to the C-terminus of HyNaC2, HyNaC3, and HyNaC5. The tags were separated from the coding sequence by a flexible linker (GGSGGGSG). Fusion was performed in a two-step PCR protocol. For HyNaC2, the forward primer was 5’-GTGGATCCGAGCTCAGGTATGAAAGC-3’, and the reverse primers were 5’-GCGTAATCTGGAACATCGTATGGGTAACCACTTCCACCACCACTTCCACCTGATTTCTTTTTGACAAACGCAGGAGC-3’ and 5’-GCTATTGTCTTCTTAAGCGTAATCTGGAACATCGTATGGGTAAGCGTAATCTGGAACATCGTATGGGTAAGCGTAATCTGGAACATCGTATGGG-3’. For HyNaC3, the forward primer was 5’-CTAGTGGATCCTAACAATGTTAAACTTCC-3’, and the reverse primers were 5’-AGCGTAATCTGGAACATCGTATGGGTAACCACTTCCACCACCACTTCCACCTTTAGTCGTAAATTTTTCAAAAAATCTGGTGTAGAG-3’ and 5’-GCTTGGTACCTTAAGCGTAATCTGGAACATCGTATGGGTAAGCGTAATCTGGAACATCGTATGGGTAAGCGTAATCTGGAACATCGTATGGG-3’. And for HyNaC5, the forward primer was 5’-TAGTGGATCCGAGCTCGATTAAAAATGC-3’, and the reverse primers were 5’-GCGTAATCTGGAACATCGTATGGGTAACCACTTCCACCACCACTTCCACCAGATTGAATTCTCAATAGTTGGTGATACAC-3’ and 5’-GCTTGGTACCTTAAGCGTAATCTGGAACATCGTATGGGTAAGCGTAATCTGGAACATCGTATGGGTAAGCGTAATCTGGAACATCGTATGG-3’. Constructs were cloned into pcDNA3.1(-) using BamHI and BbsI (HyNaC2) or BamHI and KpnI (HyNaC3 and HyNaC5). The sequences of the constructs were confirmed by sequencing of the full coding sequence (Eurofins Genomics).

Codon optimization of HyNaC2, HyNaC3, and HyNaC5 to *Homo sapiens* codon usage preferences was performed by BioCat GmbH (Heidelberg, Germany). The cDNA was synthesized with a C-terminal flexible linker (GGSGGGSG) followed by three HA-tags and cloned into pcDNA3.1(-) using the same restriction sites as above. To create the codon-optimized constructs without linkers and tags, PCR was performed to insert an additional restriction site of either BbsI (HyNaC2) or KpnI (HyNaC3 and HyNaC5) between the end of the HyNaC coding sequence and the start of the linker. The resulting constructs were digested with the respective restriction enzymes and cloned into pcDNA3.1(-). The successful removal of linkers and tags was confirmed by sequencing (Eurofins Genomics).

### Cell culture

Cells were grown at 37 °C in a humidified atmosphere with 5% CO_2_. HEK 293 T and COS-7 cells were maintained as an adherent monolayer in Dulbecco’s modified Eagle’s medium supplemented with 10% FBS and 2 mM L-glutamine. Cells were passaged every 2–3 days.

### Electrophysiological recordings

Cells were transfected with 0.6 µg each of HyNaC2, HyNaC3, and HyNaC5 plus 0.2 µg of GFP per 35 mm dish using calcium phosphate transfection. Approximately 48 h after transfection, the culture dish with attached cells was mounted on the stage of an inverted phase-contrast microscope (IX71, Olympus). The recording chamber was perfused with the following bath solution (in mM): NaCl 128, KCl 5.4, HEPES 10, glucose 5.5, MgCl_2_ 1, CaCl_2_ 2; pH was adjusted with NaOH to 7.4 at RT (22–25 °C).

Patch-clamp experiments were performed in the whole-cell configuration, using an Axon-200B amplifier (Molecular Devices; San Jose, CA, USA) and an Axon Digidata 1440 A acquisition system controlled by the Clampex 10.0 software (Molecular Devices). Signals were low pass filtered at 1 kHz and digitized at 4 kHz. Micropipettes (4–6 MΩ) were prepared from borosilicate glass capillaries with a micropipette puller (DMZ-Universal Electrode Puller; Zeitz Instruments, Martinsried, Germany) and filled with an intracellular solution containing (in mM): NaCl 10, KCl 121, HEPES 10, EGTA 5, MgCl_2_ 2. Holding potential was −70 mV. Capacitance and series resistances were compensated electronically at 80% and digital data were stored in a compatible PC for off-line analysis using Clampfit 10.0 software (Molecular Devices).

### Ca^2+^ imaging

For Ca^2+^ imaging, cells were grown on coverslips and transfected as described for electrophysiological recordings. Approximately 48 h after transfection, cells were loaded with 2 µM of Fura-2-AM (Molecular Probes) for 30 min at 37 °C. Subsequently, the coverslips were mounted in a cell chamber and perfused with bath solution. Fura-2 was excited at 340/380 nm and the emission was recorded between 470 and 550 nm on an inverted microscope (IX71, Olympus, Chromaphor) using a sensicam CCD camera (PCO imaging). Acquisition and data analysis were performed using Till Vision real-time imaging software (Till Photonics). At the end of each experiment, a bath solution containing 1 µM ionomycin was used as a positive control.

### Immunoblotting

Cells were transfected with 0.66 µg each of HyNaC2, HyNaC3, and HyNaC5 in a 35 mm dish using 6 µl of 1 mg/ml polyethylenimine (PEI) aqueous solution as transfection reagent. The transfection medium was replaced with normal medium the next day. Approximately 48 h after transfection, cells were washed twice with PBS and lysed in 150 µl sample buffer (50 mM Tris-HCl (pH 6.8), 2% SDS, 0.01% bromphenol blue, 10% glycerol, 100 mM DTT). Samples were sonicated 8–12 times (Sonifier 150D, Branson, USA) and heated at 65 °C for 10 min. For glycosylation experiments, cells were washed twice in PBS and scraped in 0.5 ml PBS. After adding 0.5 ml of PBS, cells were spun for 5 min at 1,000 rpm, 4 °C; the supernatant was discarded. The resulting pellet was resuspended in 150 µl ice-cold HEPES lysis buffer (150 mM NaCl, 10% Glycerol, 1% Triton X-100, 1.5 mM MgCl_2_, 1 mM EGTA, 1 mM DTT, 2x Protease Inhibitor; pH 7.5) and lysed on ice for 1–1.5 h with vortexing in between. After spinning for 10 min at 13,000 rpm, 4 °C, the resulting supernatant was used for either control, PNGase F or Endo H digest (both New England Biolabs, United States) according to the manufacturer’s protocol. Final samples were diluted in Laemmli sample buffer. 30 µl per sample was loaded onto an SDS-PAGE gel, separated, and transferred to PVDF membranes. The membranes were blocked in a Tris-buffered saline with 0.1% Tween-20 (TBST) containing 5% milk for 30 min followed by incubation with anti-HA antibody (1:1,000; Roche, Germany) in TBST with 5% milk for 1 h, and with goat anti-rat antibody conjugated to HRP (1:10,000; Jackson ImmunoResearch, United Kingdom) in TBST as a secondary antibody for 30 min. Membranes were washed 3 × 10 min with TBST after each antibody incubation step. Detection was performed with Clarity Western ECL Substrate (Bio-Rad, USA) and the resulting band intensity was measured with the image analyzer Chemi-Smart 5000 (Vilber Lourmat, Germany).

### Data analysis and statistics

Densitometric analysis of immunoblots was performed with ImageJ (https://imagej.nih.gov/ij/index.html); only bands with an apparent molecular weight between 50 and 75 kDa, corresponding to full-length HyNaC subunits, were analyzed.

Electrophysiological data were analyzed offline using Clampfit 10.0 (Molecular Devices). Current density was obtained by dividing the maximal current amplitude (pA) by cell capacitance (pF). Data are presented as mean ± S.D., except for data in Figure 5, which are shown as mean ± S.E.M. The concentration–response curve for RFamide-II and codon-optimized HyNaCs was fitted to a Hill function:

$$I=I_{min}+\;\left(I_{max}-I_{min}\right)/\left(1\;+\;{\left(\left[X\right]/EC_{50}\right)}^{H}\right)$$

where *I* is the current, [X] is the peptide concentration, *I*_min_ and *I*_max_ are the maximal and the minimal currents, respectively, EC_50_ is the concentration at which 50% of the maximal current is obtained, and *H* is the Hill coefficient (GraphPad Prism 6.0).

Statistical analysis was done using an unpaired *t-test* to compare two conditions and a one-way ANOVA (GraphPad Prism 6.0) for more than two conditions. Statistical significance was defined as *p < 0.05.*

## Results

### Native HyNaC2/3/5 is inefficiently expressed in mammalian cells

**Figure 1.**
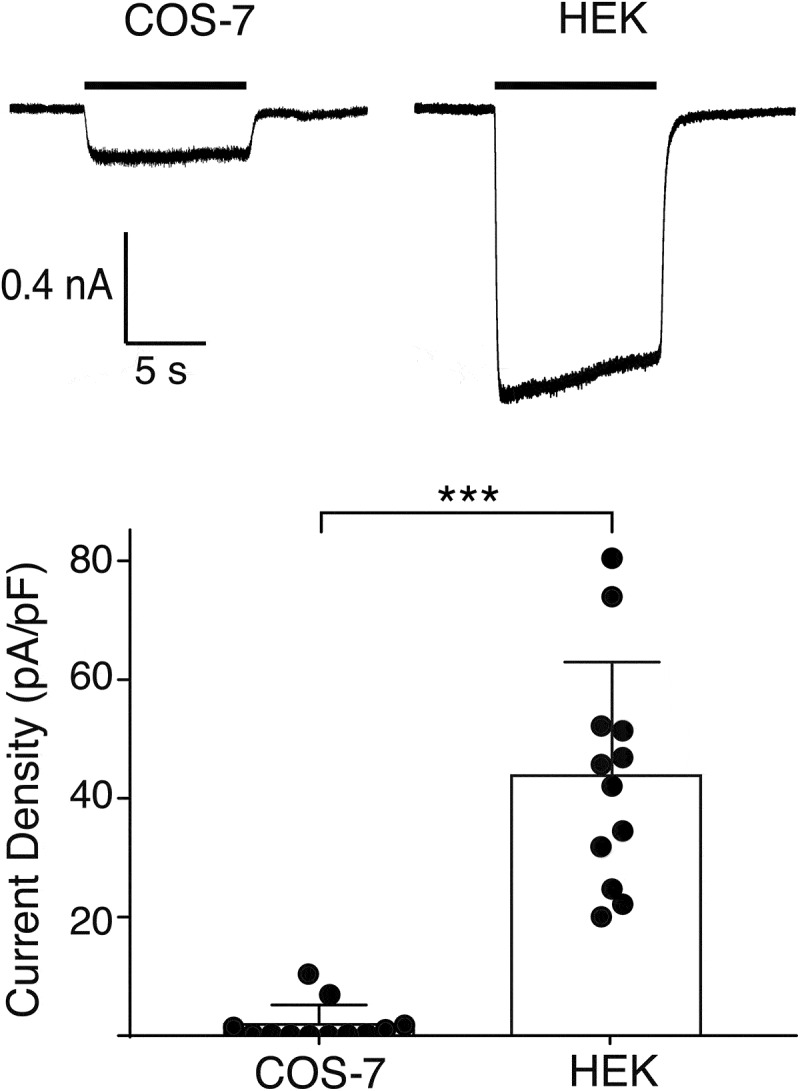
Functional expression of HyNaC2/3/5 in COS-7 and HEK 293 cells. Top, representative currents elicited by applying 10 µM RFamide I (black bar) to COS-7 cells (left) or HEK cells (right) expressing HyNaC2/3/5; both cells had similar capacitances (~20 pF). Bottom, current densities for COS and HEK cells (mean ± SD); n = 12 for each cell line; *p* < 0.0001

We transiently expressed HyNaC2/3/5 in two different mammalian cell lines, human embryonic kidney (HEK 293) cells and monkey COS-7 cells, and compared whole-cell currents evoked by Hydra-RFamide I (pQWLGGRFamide), a *Hydra* neuropeptide [4] that activates HyNaC2/3/5 in *Xenopus* oocytes; we used a concentration of Hydra-RFamide I that elicits a maximal response in oocytes (10 µM) [3]. In HEK cells, 10 µM RFamide I elicited sustained inward currents, which are typical of HyNaCs [6]; their amplitude was small, however (current density = 42 ± 10 pA/pF, n = 12; Figure 1). In COS cells, RFamide I elicited similar currents of even smaller amplitudes (5 ± 2 pA/pF; *p* < 0.001, n = 12; Figure 1).

### Codon optimization strongly increases expression of HyNaC2/3/5 in HEK cells

**Figure 2.**
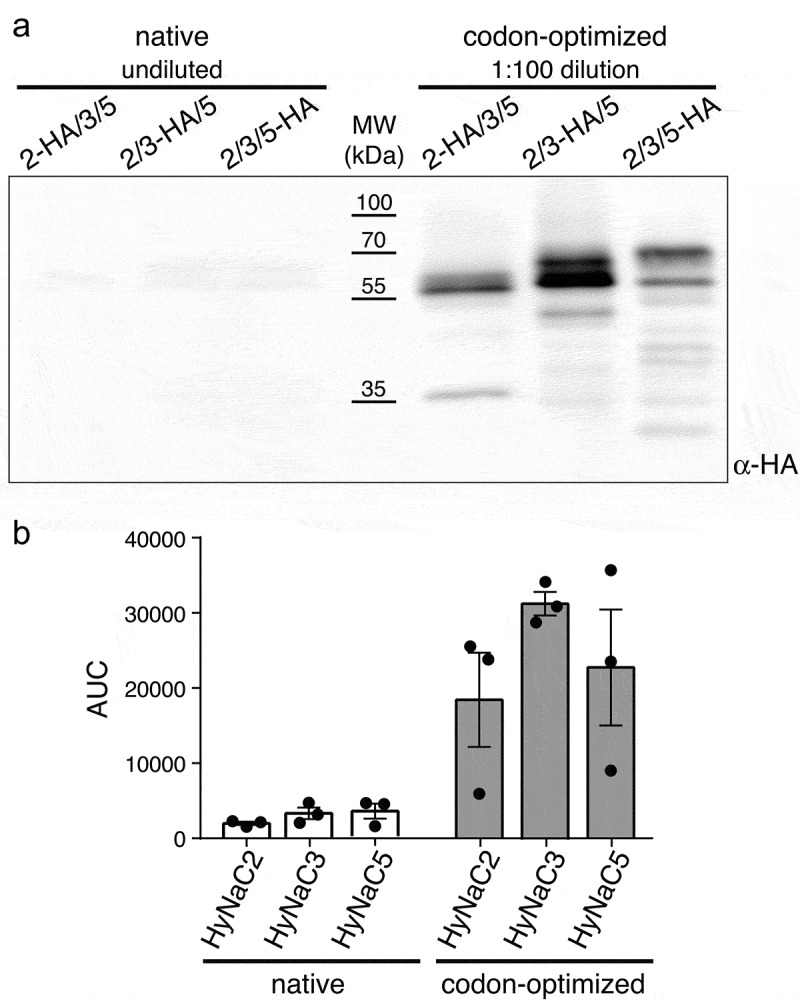
Western blot analysis of native and codon-optimized HyNaCs. A) Western blot representative for three independent experiments. Cells had been co-transfected with HyNaC2, HyNaC3 and HyNaC5; one of the subunits carried an HA-tag as indicated. Note that for optimized HyNaCs samples had been diluted 100-fold. B) Densitometric analysis of bands in the range of 50–75 kDa quantifies the increased expression of codon-optimized HyNaCs. AUC, area under the curve

Transient co-expression of HyNaC2, HyNaC3 and HyNaC5 in HEK cells resulted in very faint signals in western blots (Figure 2a, left), confirming that HyNaC2/3/5 is not efficiently expressed in HEK cells. Previously, the expression of GFP from the cnidarian organism *Aequorea victoria* could be increased 40- to 120-fold by codon optimization [7]. The Codon Adaptation Index (CAI) measures the extent to which the codon usage in a gene represents the codon preferences of the host species [8] and is one useful tool for predicting the success of heterologous gene expression. A value of 1 denotes optimal codon usage according to codon preferences of a host. The CAI values for HyNaC2, HyNaC3 and HyNaC5 vary between 0.64 and 0.66 (Table 1), similar to GFP (CAI = 0.60) [9], suggesting that codon optimization could potentially increase expression of HyNaCs in mammalian cells. For comparison, the CAI values for acid-sensing ion channels (ASICs), mammalian DEG/ENaCs that can be efficiently expressed in mammalian cells including COS-7 cells [10–12], range from 0.80 to 0.83 (Table 1). We, therefore, synthesized HyNaCs with unaltered amino acid sequence but with their codons adjusted to codon preference of *Homo sapiens*. Table 1 shows a comparison of CAI values and GC content before and after codon optimization.

**Table 1.** Codon Adaptation Index (CAI) and GC Content for native HyNaC and human ASIC subunits and for codon-optimized HyNaCs

Subunit	CAI		GC Content	
	native	optimized	native	optimized
HyNaC2	0.64	0.97	0.33	0.54
HyNaC3	0.66	0.97	0.34	0.53
HyNaC5	0.66	0.97	0.34	0.54
ASIC1a	0.83		0.55	
ASIC2a	0.81		0.52	
ASIC3	0.80		0.58

Codon-optimized HyNaC2, HyNaC3 and HyNaC5 were co-expressed in HEK cells and the individual subunits were detected via C-terminal HA-tags. Western blotting revealed a strongly increased expression of all three HyNaC subunits after codon optimization (Figure 2a). In fact, expression was so strongly increased that samples had to be diluted prior to SDS-PAGE to allow comparison of their expression with that of native (non-codon-optimized) HyNaCs on the same blot. A densitometric comparison of the expression of native and optimized HyNaC constructs showed a > 9-fold increase for HyNaC2, a > 9-fold increase for HyNaC3 and a > 6-fold increase for HyNaC5 (Figure 2b, n = 3). Since samples of codon-optimized HyNaCs had to be diluted 100-fold, it is difficult to estimate the true increase in expression, which is likely to be higher than the values reported here.

Codon-optimized HyNaCs showed two prominent bands in Western blots, one at their predicted molecular weight of 54–55 kDa and one at a slightly higher apparent molecular weight (Figure 2a). We will show below that the lower band likely corresponds to the unglycosylated form of HyNaC subunits and the higher band to the glycosylated form. Prominent expression of the unglycosylated forms indicates a large pool of immature HyNaC subunits after codon optimization. Similarly, we observed a variable amount of low molecular weight antigens in immunoblots, which suggest that part of the HyNaC pool was proteolytically cleaved.

### Electrophysiological characterization of HyNaC2/3/5 in HEK cells

**Figure 3.**
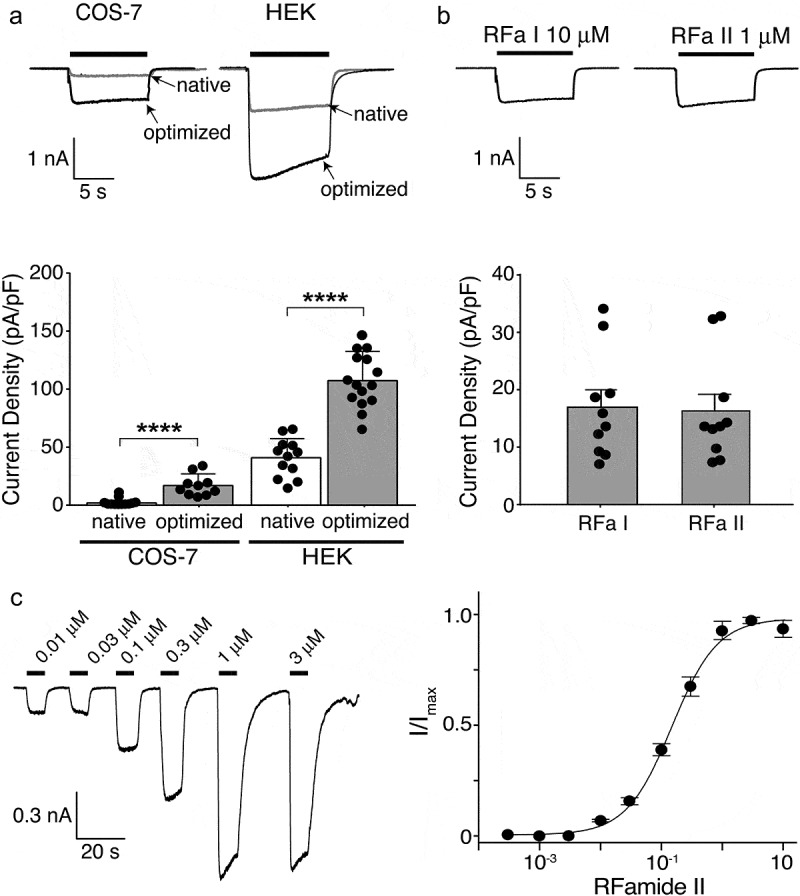
Electrophysiological characterization of codon-optimized HyNaC2/3/5 in HEK 293 cells. A) Top, representative current traces of native (gray trace; current elicited by 10 µM RFamide I) and codon-optimized (black trace; current elicited by 1 µM RFamide-II) HyNaC2/3/5 expressed in COS-7 cells (left) or HEK cells (right); all cells had similar capacitances (~20 pF). Bottom, current densities of native and codon-optimized HyNaC2/3/5 (mean ± SD). n ≥ 10 for each cell line; ****, p < 0.0001 (unpaired t-test). Data for native HyNaC expressed in COS-7 cells are from Figure 1. B) Top, representative currents of codon-optimized HyNaCs expressed in COS-7 cells elicited by 10 µM RFamide I or 1 µM RFamide II. Bottom, current densities after activation with the two peptides (mean ± SD). n = 10, p = 0.87 (unpaired t-test). C) Left, representative currents of codon-optimized HyNaC2/3/5 elicited by different concentrations of RFamide II. Right, concentration–response curve. Error bars represent S.D. The line represents a fit to the Hill equation. n = 8

We next analyzed the functionality of the codon-optimized channels in COS-7 and HEK 293 cells using a whole-cell patch clamp. 1 µM Hydra-RFamide II (pQWFNGRFamide), a concentration that elicits a maximal response in oocytes [3], elicited sustained inward currents of significantly larger amplitude than for the channel encoded by the native genes in both COS-7 (17 ± 9 pA/pF, n = 10, versus 2 ± 3 pA/pF, n = 12; p < 0.001; Figure 3a) and HEK cells (113 ± 9 pA/pF, n = 15, versus 40 ± 6 pA/pF, n = 12; p < 0.001; Figure 3a), but current amplitude in COS-7 cells was still small. We verified that activation with 1 µM Hydra-RFamide I and 10 µM Hydra-RFamide II yielded comparable current amplitudes in COS-7 cells (Figure 3b). When expressed in HEK cells, the apparent affinity for RFamide II was 0.14 ± 0.03 µM (Figure 3c), similar to the apparent affinity of HyNaC2/3/5 expressed in oocytes [3]. Thus, codon optimization allowed the functional characterization of HyNaC2/3/5 in mammalian cells. Of note, the increase in the current density (approximately 3-fold) was not as strong as the increase in total protein estimated by Western blotting (Figure 2), suggesting that only part of the additional HyNaC subunits reached the plasma membrane, which is consistent with the presence of a relatively high abundance of immature HyNaC subunits after codon optimization (Figure 2).

In *Xenopus* oocytes, activation of HyNaCs elicits biphasic currents with a transient peak and a sustained inward current [1,3]. It has been shown that the transient peak current is mediated by the secondary activation of Ca^2+^-activated Cl^–^channels that are endogenous to ooyctes and get activated by Ca^2+^ influx through HyNaCs. Only the sustained current is directly mediated by HyNaCs [2,6]. The monophasic sustained currents in HEK and COS-7 cells shown here are consistent with this finding.

**Figure 4.**
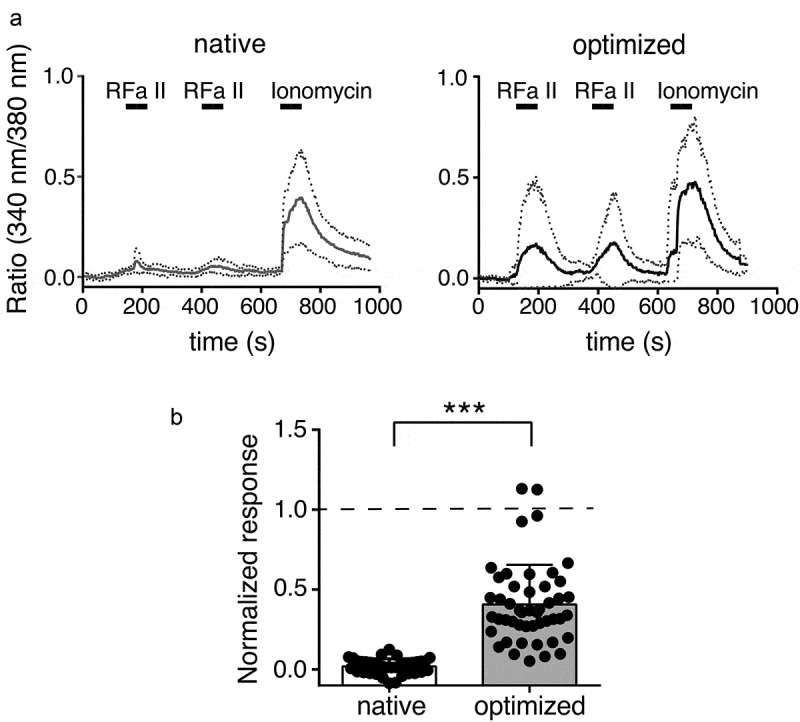
Ca^2+^ responses of HEK 293 cells expressing native and codon-optimized HyNaC2/3/5. A) Mean Ca^2+^ responses of 10 cells to stimulation with 1 µM Hydra-RFamide II (RFa II) or 1 µM ionomycin. Responses are presented as ratio of 340/380 nm fura-2 fluorescence; basal fluorescence was subtracted. Dotted lines represent the SD. B) Quantification of 6 experiments similar to the one shown in A from 2 different transfections (mean ± SD; ***, p < 0.001, unpaired t-test). For individual cells, the mean of the two Ca^2+^ responses to stimulation with Hydra-RFamide II was normalized to the response to ionomycin. The total number of cells analyzed was n = 36 for native HyNaCs and n = 48 for codon-optimized HyNaCs

To detect Ca^2+^ influx through HyNaCs in HEK cells, we performed Ca^2+^ imaging. Whereas the application of 1 μM Hydra-RFamide II to HEK cells expressing native HyNaC2/3/5 hardly increased intracellular Ca^2+^ above background, the same peptide application to HEK cells expressing codon-optimized HyNaCs elicited robust intracellular Ca^2+^ signals (Figure 4).

### A C-terminal HA-tag reduces HyNaC current amplitude

**Figure 5.**
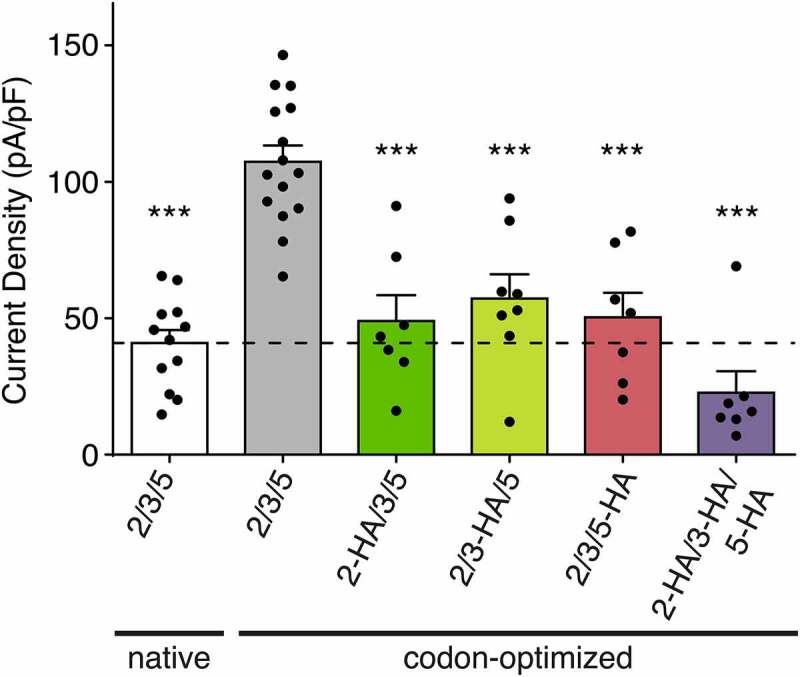
Effect of the HA-tag on HyNaC current density. Current densities of native HyNaC2/3/5 (white bar), codon-optimized HyNaC2/3/5 (gray bar) and codon-optimized HyNaC2/3/5 with one or more subunits carrying an HA-tag (green, yellow, pink and purple bars). Error bars represent S.E.M; n = 7 to 15; *** p < 0.0001 (one-way ANOVA)

It has been reported that different epitope-tags can produce unexpected effects on the ion channel expression and function [13]. To assess the effect of the HA-tags on the functional properties of HyNaC subunits, we applied RFamide II (1 µM) on HyNaC2/3/5 containing one or three tagged, codon-optimized subunits. We found that one HA-tag significantly reduced the current amplitude, irrespective of which subunit carried the tag (49 ± 9 pA/pF when HyNaC2 carried the tag, 57 ± 8 pA/pF when HyNaC3 carried the tag and 50 ± 9 pA/pF when HyNaC5 carried the tag versus 84 ± 9 pA/pF when no subunit carried the tag; n = 7–8; *p* < 0.05). Consistently, codon-optimized channels with all subunits carrying an HA-tag further reduced HyNaC2/3/5 current amplitudes to 22 ± 7 pA/pF, n = 7 (*p* < 0.0001; Figure 5).

### HyNaCs are complex-glycosylated in HEK cells

**Figure 6.**
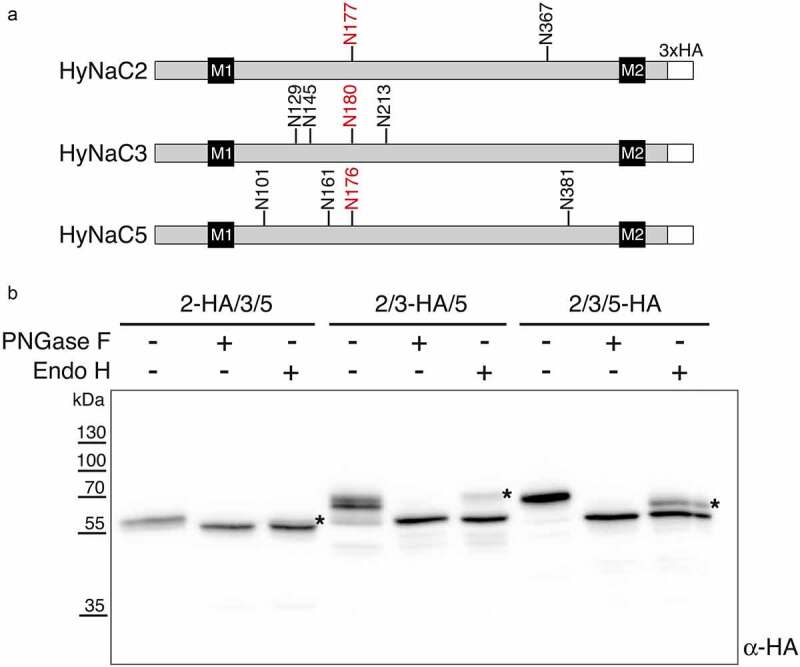
Western blot analysis of glycosylation of codon-optimized HyNaCs. A) Linear diagrams of HyNaC2, HyNaC3, and HyNaC5 illustrating the predicted positions of N-glycans, transmembrane domains M1 and M2, and the C-terminal HA epitopes. The one conserved glycan is marked in red. B) Treatment with PNGase F or Endo H reduced the molecular mass of all HyNaC subunits, indicating N-glycosylation. Stars indicate the endo-H resistant forms. Cells had been co-transfected with HyNaC2, HyNaC3 and HyNaC5; one of the subunits carried an HA-tag as indicated. Western blot is representative for three independent experiments

Lastly, we used the improved expression levels of codon-optimized HyNaCs to explore their glycosylation. For this, we used PNGase F, which removes all N-glycans, and endoglycosidase H (Endo H), which cleaves only non-complex glycans. HyNaC2 has two consensus sequences for N-linked glycosylation (N-X-S/T, where X is any amino acid except P) in its extracellullar domain (ECD), while HyNaC3 and HyNaC5 have four (see Figure 6a). As shown in Figure 6b, PNGase F reduced the apparent molecular weight of all three HyNaCs to their predicted molecular weight of 54–55 kDa, revealing that they all carry N-glycans. The reduction in molecular weight was approximately 3 kDa for HyNaC2 and approximately 9 kDa for HyNaC3 and HyNaC5, suggesting that all consensus sequences are indeed glycosylated. Treatment with Endo H reduced a substantial fraction of the HyNaC pool to the size of the fully deglycosylated proteins (Figure 6b). But in each case, an Endo H-resistant form was also discernible, indicating that part of the total HyNaC pool carried complex N-glycans in HEK 293 cells. Using densitometry, we estimated that ~25% of HyNaC2, ~15% of HyNaC3, and ~35% of HyNaC5 were complex glycosylated.

## Discussion

Different organisms display nonrandom bias for synonymous codons based on their specific population of tRNAs [14]. Biased codon usage has been characterized in various species, both prokaryotic and eukaryotic [15–18]. Limited availability of cognate tRNAs for rare codons can slow or even limit protein production from heterogeneous genes in host organisms. High expression levels of a gene correlate with the usage of more frequent codons [19,20] while less preferred codons appear more often in genes with lower expression levels [21]. Research on codon usage preferences has subsequently led to the optimization of codons of a foreign gene to the preferences of the host organism to improve total protein expression levels. Various studies have shown that heterologous expression of eukaryotic genes in prokaryotic expression systems such as *E. coli* can be improved up to 40-fold with regard to total protein levels [22,23]. The adjustment of codons in prokaryotic genes for expression in mammalian cells has demonstrated to successfully increase expression as well [24]. Lastly, heterologous expression of viral proteins was improved similarly [25] and can be used to develop better suited immunogens against viral infections such as hepatitis C [26].

The first examples of successful codon optimization to increase the expression of a membrane protein in mammalian cells were the two subunits of the glutamate-gated Cl^−^ channel from *C. elegans*, GluClα1 and GluClβ [9]. Concerning cnidarian ion channels, codon optimization had previously been successfully used for the functional characterization in human HEK 293 cells of the TRPM2 ion channel from the sea anemone *Nematostella vectensis* [27]. Our study demonstrates that codon optimization strongly increases the total expression of the cnidarian peptide-gated ion channel HyNaC2/3/5 in HEK 293 cells and substantially increases functional channels on the cell surface.

CAI of HyNaCs was >0.9 after codon optimization. We would like to note, however, that it has been reported that rare codons at some sequence positions might lead to local pauses in translation, enhancing cotranslational protein folding and increasing the likelihood of forming the native protein structure [28,29]. Therefore, CAI values of >0.9 might boost translation but potentially constrain the folding of HyNaC subunits into their native conformation. Inefficient folding of HyNaC subunits could explain the relatively high abundance of immature proteins in immunoblots (Figure 2) and the smaller increase in the current density than protein abundance after codon optimization.

In contrast to mammalian cells, GluClα and GluClβ, can be readily expressed in *Xenopus* oocytes [30]. This situation is reminiscent of HyNaCs, where the channels with native codons give rise to large current amplitudes in *Xenopus* oocytes [2,3]. Similarly, voltage-gated K^+^ channels from the hydrozoan jellyfish *Polyorchis penicillatus* and from *N. vectensis*, as well as voltage-gated Na_v_-like channels, Erg K^+^ channels and HCN channels from *N. vectensis* were all successfully expressed and functionally characterized in *Xenopus* oocytes [31–35]. This indicates that *Xenopus* oocytes are a permissive expression system that does not require codon optimization for the expression of cnidarian ion channels. Notably, a DEG/ENaC from the placozoan *Trichoplax adhaerens*, a species that diverged early in the evolution of multicellular animals and is sister to cnidarian as well as bilaterian animals, has been successfully expressed in CHO cells without codon optimization [36].

Studying cnidarian ion channels has great relevance for neuroscience [37]. Increased expression of HyNaC2/3/5 will facilitate its biochemical and functional characterization in mammalian cells. We demonstrate this by showing that activation of HyNaC2/3/5 elicits intracellular Ca^2+^ signals and by analyzing the glycosylation of HyNaC2, HyNaC3 and HyNaC5 in HEK 293 cells. Because HyNaCs are expressed on epitheliomuscular cells of *Hydra* [1,2] and have been implicated in neuromuscular transmission [2,3], their uniquely high Ca^2+^ permeability could allow sufficient Ca^2+^ influx to induce muscle contraction [5]. As we now show, their efficient expression in mammalian cell lines allows to test these questions experimentally. Moreover, it is conceivable to develop peptide-gated ion channels as tools to manipulate mammalian neurons, for example by their native peptide ligands, which are not present in mammalian nervous systems, or by small-molecule agonists, or by tethered, photoswitchable peptide ligands [38]. Due to their sustained currents and high Ca^2+^ permeability [2,6], HyNaCs might be particularly interesting for such applications. Their efficient expression in mammalian cells shown here is a first step in this direction.
